# Systematic review of the role of anti-inflammatory treatments in animal models of venous thromboembolism

**DOI:** 10.1016/j.jvssci.2026.100450

**Published:** 2026-09-21

**Authors:** Matti Jubouri, Thomas Brankin-Frisby, Alexander S. Grover, Benedict RH. Turner, Alun H. Davies, Alberto Smith

**Affiliations:** a Section of Vascular Surgery, Department of Surgery and Cancer, Imperial College London, London, UK; b Nuffield Department of Population Health, University of Oxford, Oxford, UK; c Royal Free Hospital, Royal Free London NHS Foundation Trust, London, UK; d Academic Department of Vascular Surgery, St Thomas's Hospital, Kings College London, London, UK

**Keywords:** Anti-inflammatory, Deep vein thrombosis, Fibrosis, Post-thrombotic syndrome, Scarring, Venous thromboembolism

## Abstract

**Objective:**

The study aimed to systematically appraise the impact of anti-inflammatory pharmacological treatments on thrombosis, inflammation, and fibrosis parameters in animal models of venous thromboembolism (VTE).

**Methods:**

A systematic review was conducted according to Preferred Reporting Items for Systematic Reviews and Meta-Analyses guidelines with a registered protocol (PROSPERO: CRD42023456793). Free-word and medical subject heading terms were used to search the Medline, Embase, and Cochrane databases for eligible articles. All studies and conference abstracts reporting the use of an anti-inflammatory agent in an animal model of VTE were included. The primary efficacy outcome was the impact of anti-inflammatory drugs on post-thrombotic venous fibrosis and thrombus burden. The secondary outcome was the impact of anti-inflammatory drugs on measures of inflammation. Quality scoring and risk of bias assessment were conducted using the SYRCLE tool.

**Results:**

A total of 42 studies were included in the final review, but fibrosis as an outcome measure was generally poorly reported (seven studies). Although meta-analysis was planned, heterogeneity in species, study design, and intervention precluded it. Overall, selectin inhibitors demonstrated the greatest sustained impact on post-thrombotic fibrosis (three studies), followed by statins (two studies) and the antimitotic agent colchicine (one conference abstract). Both selectin inhibitors and statins have consistently yielded positive results, reducing vein scarring, alleviating thrombus burden, and inhibiting inflammation. The quality of evidence was generally poor.

**Conclusions:**

Inflammation involves various evolutionarily conserved mechanisms that cause tissue damage and fibrosis, but fibrosis is generally poorly studied in preclinical models of VTE. Future studies should systematically evaluate the efficacy of anti-inflammatory agents initiated after thrombosis to better mimic clinical scenarios.

## Background

Venous thromboembolism (VTE) remains a pertinent hospital-acquired complication and is the third leading cardiovascular disease in incidence and mortality.^1^ Consisting of deep venous thrombosis (DVT) and pulmonary embolism (PE), VTE has a lifetime incidence of 2% to 5%.^2^ The global burden of VTE was recognized in 2008 with the U.S. Surgeon General's call to action to innovate treatments to prevent and treat VTE.^3^ Morbidity from DVT is particularly prevalent, with 20% to 50% of patients developing post-thrombotic syndrome (PTS).^4^ This syndrome, resulting from venous scarring, consists of leg pain, swelling, venous claudication, and skin changes, with progression to ulceration.^4^^,^^5^ Patients with PTS experience reductions in quality of life comparable with those with heart failure or cancer.^6^^,^^7^

Inflammation is critical for the evolution and resolution of thrombosis, but it also activates the wound healing program, leading to tissue repair through scarring (fibrosis).^8^^,^^9^ Fibrosis is characterized by the deposition of excessive, disorganized extracellular matrix and the differentiation, proliferation, and activation of myofibroblasts, which results in altered tissue properties such as increased stiffness and reduced compliance.^10^ Patients with PTS demonstrate changes typical of fibrosis, including a thickened, stiff vein wall, excessive collagen deposition, myofibroblasts, neointima formation, and intimal hyperplasia.^11^, ^12^, ^13^, ^14^, ^15^ These patients also present with elevated inflammatory markers, such as C-reactive protein and interleukin-6 (IL-6).^9^^,^^16^^,^^17^ The release of proinflammatory cytokines, such as IL-6, drives the acute inflammatory process and has additional effects on fibrosis, including the upregulation of profibrotic cytokines (eg, transforming growth factor beta), synthetic vascular smooth muscle cell phenotypes, increased extracellular matrix protein deposition, and fibroblast proliferation/myofibroblast differentiation.^18^, ^19^, ^20^, ^21^, ^22^, ^23^ The ensuing venous fibrosis perturbs normal vascular architecture and function: inflammation damages the venous valve structure and causes vascular stiffness from scar tissue, thereby restricting vessel compliance.^24^^,^^25^ Intraluminal scarring can also occur, causing venous obstruction that reduces blood outflow, leading to venous claudication, stasis, and ulceration.^25^^,^^26^ Ongoing proinflammatory activation increases the risk of both VTE and recurrent VTE.^27^^,^^28^ Thus, post-thrombotic inflammation may contribute to the development and recurrence of VTE, as well as fibrotic complications such as PTS and chronic thromboembolic pulmonary hypertension.

Clinically, venous fibrosis is a critical determinant of long-term outcomes in patients with DVT, directly contributing to the development of PTS. Fibrotic vein wall changes, such as stiffening and luminal narrowing, not only impair venous return but also predispose patients to chronic venous hypertension, pain, and ulceration. Thus, targeting fibrosis is not only mechanistically relevant but also potentially therapeutic in reducing the burden of post-thrombotic complications.

Current clinical guidelines suggest that patients with iliofemoral DVT may derive the greatest benefit from catheter-directed or mechanical thrombus removal strategies; however, the role of these interventions in other venous territories continues to be investigated. In addition, morbidity from VTE-related complications remains high.^29^ Anticoagulation reduces rates of VTE recurrence but does little to impact rates of post-thrombotic scarring and subsequent PTS. One approach to reduce venous scarring could be targeting inflammation; however, there are no human studies, and the safety evidence from preclinical models has not been previously described. This review will outline the evidence for anti-inflammatory pharmacologic treatments to impact thrombosis, inflammation, and fibrosis parameters in animal models of VTE.

## Methods

This review was conducted according to the Collaborative Approach to Meta-Analysis and Review of Animal Data from Experimental Studies (SYRCLE) guidelines and the Preferred Reporting Items for Systematic Reviews and Meta-Analyses (PRISMA) guidelines.^30^^,^^31^ A registered protocol (PROSPERO: CRD42023456793) was used to screen the online databases MEDLINE, EMBASE, and the Cochrane Library up to July 3, 2023.

### Search strategy

Searches were initially carried out without restrictions regarding publication type or language. Gray literature was not screened because this was a review of preclinical studies. Medical subject heading terms and free-word searches were used; the full search strategy is available in Supplementary Table (online only). References of the included articles were screened for further eligible studies. Two reviewers (T.B.-F. and A.S.G.) independently screened the identified articles against the inclusion criteria; any disputes were mediated by a third reviewer (B.R.H.T.). The screening process was conducted using Endnote X20 (Clarivate); titles and abstracts were screened before full-text review. Supplementary Table (online only) highlights the search strategy used to search the Medline, Embase, and Cochrane databases.

### Eligibility criteria

Included articles had the following characteristics:

1. Articles testing drugs with an inhibitory action on the inflammatory cascade.
2. To enhance inclusivity, experimental studies published as abstracts or peer-reviewed studies were included.
3. At least one reported outcome of interest (inflammation markers, fibrosis, or thrombosis parameters).

Excluded articles had the following characteristics:

1. Therapies designed to exacerbate inflammation.
2. Studies exclusively involving heparin, low-molecular-weight heparins (LMWHs), or direct oral anticoagulants (hereafter referred to antithrombotic therapies) because these are outside the scope of this review. However, studies administering these in combination with anti-inflammatory therapy were eligible for inclusion.
3. Case reports, letters, or opinion pieces.
4. Studies not reporting a prespecified outcome.

### Data extraction and analysis

Data from the included articles were extracted using a standardized template in Microsoft Excel by two reviewers (T.B.-F. and A.S.G.) independently. Any discrepancies were reviewed and mediated by a third reviewer (B.R.H.T.). Because of significant heterogeneity in species, drugs, and dose, a narrative systematic review was performed without meta-analysis.

### Outcomes

The primary efficacy outcome was the impact of anti-inflammatory drugs on post-thrombotic venous fibrosis and thrombus burden. The secondary outcome was their impact on measures of inflammation, such as inflammatory markers, leukocyte recruitment, and vein wall remodeling, to determine the mechanism by which these drugs may exert their antifibrotic effect.

### Quality and risk-of-bias assessment

The SYRCLE tool was used to assess the quality of evidence and risk of bias.

## Results

A total of 6063 abstracts were obtained from database searches. After removing duplicates, 4843 discrete articles were screened against the eligibility criteria. Title and abstract screening identified 77 articles for full-text review, of which 43 studies were included in the final review. Fig displays the full PRISMA flow diagram. Studies originated from groups in the United States, China, Brazil, and Italy. Five of the included studies were conference abstracts,^32^, ^33^, ^34^, ^35^, ^36^ whereas the remaining studies were published experimental studies. The most common animal model was mouse, (n = 18),^27^^,^^32^, ^33^, ^34^, ^35^, ^36^, ^37^, ^38^, ^39^, ^40^, ^41^, ^42^, ^43^, ^44^, ^45^, ^46^, ^47^ followed by rat (n = 10),^48^, ^49^, ^50^, ^51^, ^52^, ^53^, ^54^, ^55^, ^56^ baboon (n = 8),^57^, ^58^, ^59^, ^60^, ^61^, ^62^, ^63^, ^64^ rabbit (n = 5),^65^, ^66^, ^67^, ^68^, ^69^ and lamb (n = 1).^70^ There were 11 studies on DVT models and 33 studies on PE models. Although inflammation (n = 42) and thrombus burden (n = 35) outcomes were generally well reported, only a few included studies (n = 8) reported outcomes for venous fibrosis. The most common classes of anti-inflammatory treatments were statins (n = 10) and selectin inhibitors (n = 11) followed by glucocorticoid treatments (n = 3), nonsteroidal anti-inflammatory drugs (NSAIDs) (n = 3), antimitotic agents (n = 1), and other miscellaneous treatments. Table I contains the baseline characteristics of the included studies. Table II provides an overview of the experimental methodologies utilized in the included studies. Data on all results for fibrosis, thrombosis, and inflammation are reported in Table III.

**Fig:**
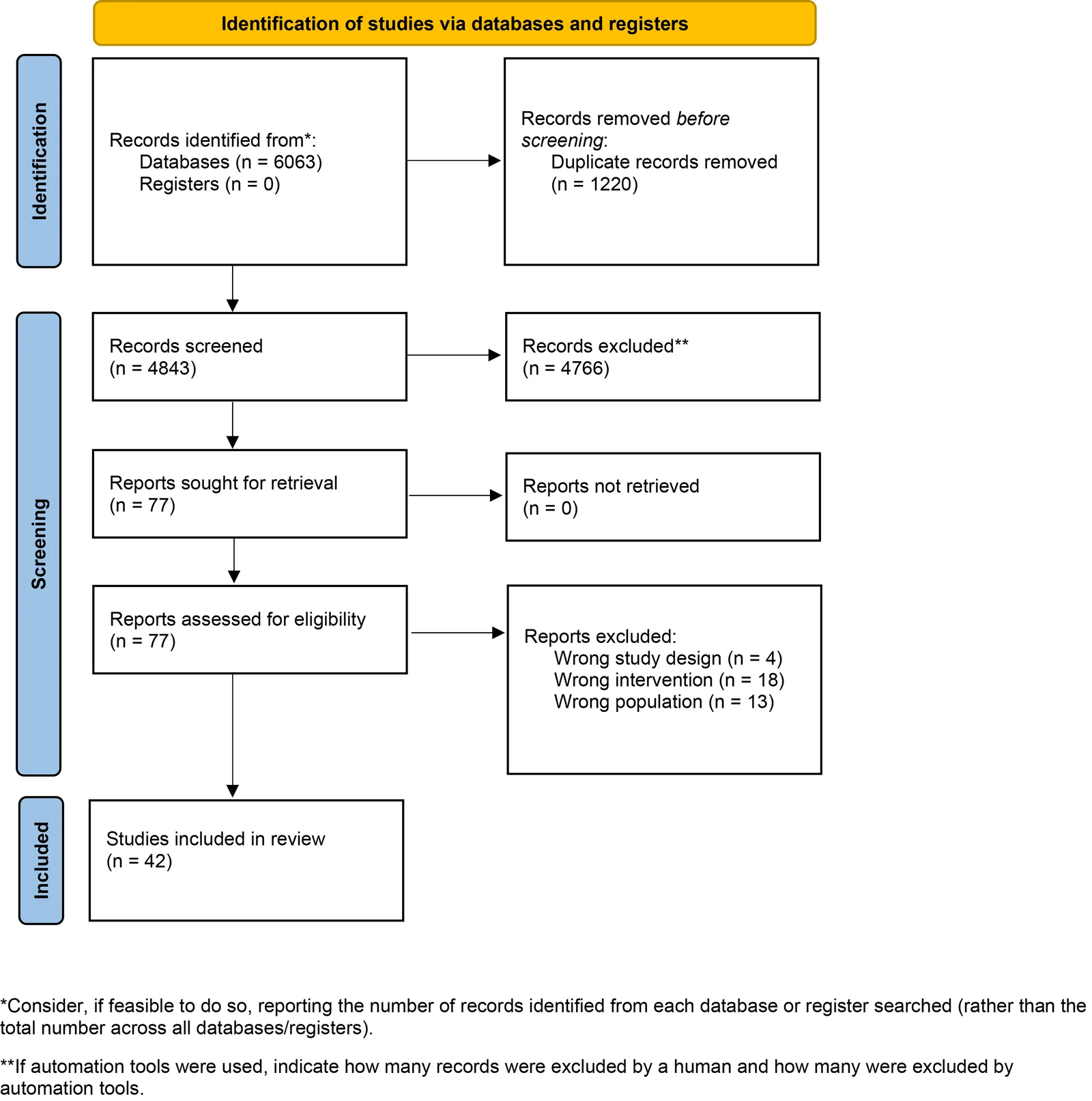
Preferred Reporting Items for Systematic Reviews and Meta-Analyses flow diagram of studies included and excluded from the review.

**Table I:** Characteristics of included animal studies evaluating anti-inflammatory therapies in experimental venous thromboembolism models

Study	Year	Species	Strain	No.	Anti-inflammatory	Class
Kessinger et al^45^	2021	Mouse	C57BL/6J	75	Rosuvastatin	Statin
Feng et al^65^	2017	Rabbit	New Zealand white rabbits	48	Simvastatin	Statin
Feng et al^66^	2016	Rabbit	New Zealand white rabbits	72	Simvastatin	Statin
Kessinger et al^44^	2015	Mouse	C57/BL6 (naive male 14-week-olds)	282	Atorvastatin and rosuvastatin	Statin
Patterson et al^37^	2013	Mice	ApoE^−/−^ mice (stock #2052, Jackson Laboratories)	180	Rosuvastatin	Statin
DeRoo et al^32^	2012	Mouse	C57BL/6	N/R	Rosuvastatin	Statin
Sun et al^67^	2011	Rabbits	New Zealand White Rabbits	40	Atorvastatin	Statin
Souza-Costa et al^48^	2007	Rats	Wistar	Different for each experiment	Atorvastatin	Statin
Neto-Neves et al^70^	2011	Lambs	Mixed-bred	22	Atorvastatin and sildenafil	Statin/NO inducer
Rossiello et al^38^	2005	Mouse	CD-1	N/R	Nitropravastatin	Statin/NO inducer
Wang et al^55^	2013	Rats	Sprague-Dawley	N/R	NO from lipidic microbubbles	Nitric oxide activation
Myers et al^59^	2022	Baboon	*Papio Anubis*	23	GMI-1271 (Uproleselan [GMI])	Selectin inhibitor
Myers et al^58^	2020	Baboon	N/R	9	GMI-1271	Selectin inhibitor
Wang et al^35^	2017	Mice	C57BL/6	60	Methotrexate	Selectin inhibitor
Diaz et al^64^	2015	Baboon	N/R	12 (divided into four groups)	ARC5692 (Anti-P-selectin aptamer)	Selectin inhibitor
Anyanwu et al^33^	2012	Mouse	C57BL/6	12 (four CO-exposed mice, four normocapnic mice, four sham-operated mice)	CO was administered for 7 days to the treated mice	Selectin inhibitor
Diaz et al^34^	2009	Mouse	C57BL/6	N/R	ARC5690	Selectin inhibitor
Bedard et al^49^	2008	Rats	Sprague-Dawley	15	PSI-697	Selectin inhibitor
Meier et al^62^	2008	Baboon	*Papio anubis*	9	P-selectin-inhibitor-421 (PSI-421)	Selectin inhibitor
Myers et al^61^	2007	Baboon	*Papio anubis*	9	PSI-697	Selectin inhibitor
Myers et al^57^	2002	Baboon	*Papio anubis*	8	rPSGL-Ig	Selectin inhibitor
Myers et al^60^	2001	Baboon	*Papio anubis*	8	rPSGL-Ig	Selectin inhibitor
Wakefield et al^63^	2000	Baboon	N/R	12	Recombinant P-selection glycoprotein ligand-Ig	Selectin inhibitor
Zhang et al^71^	2017	Rats	Sprague-Dawley	90 (divided into 9 groups)	Aspirin	NSAID
Qi et al^36^	2017	Mouse	C57BL/5	60	Colchicine	NSAID
Wang et al^56^	2013	Rats	Sprague-Dawley	108 rats in 6 groups of 18.	Aspirin	NSAID
Sun et al^72^	2020	Mouse	BALB/c	40	N/R	Steroid
Trejo et al^68^	2007	Rabbits	New Zealand white rabbits	30	Hydrocortisone and papaverine	Steroid/phosphodiesterase inhibitor
Dai et al	2022	Mouse	C57BL/6	n = 6 in each group (five groups total)	Ruscogenin	Steroidal sapogenin
Li et al^69^	2022	Rabbit	New Zealand white rabbits	10	Warfarin and EGCG	Traditional Chinese medicine
Zhou et al^43^	2020	Mouse	C57BL/6J	16 (for ligation model)	Tongji 2	Traditional Chinese medicine
Zhang et al^52^	2016	Rats	Sprague-Dawley	48 (divided into 4 groups)	Danhong Huayu Koufuye (DHK)	Traditional Chinese medicine
Myers et al^46^	2003	Mouse	C57BL/6	99	IL-10	Interleukin
Henke et al^50^	2000	Rats	Sprague-Dawley	4 in each group	IL-10	Interleukin
Li et al^42^	2016	Mouse	C57BL/6	30	Sodium hydrosulfide	Increases PECAM-1, eNOS, and iNOS expression
Chen et al^39^	2019	Mouse	C57BL/6	80	Catestatin	Catecholamine secretion inhibitory peptide
Chen et al^73^	2022	Mouse	C57BL/6	48	Ginsenoside Rg5	Anti-inflammatory agent derived from Panax ginseng species
Pan et al^40^	2021	Mice	C57BL/6 and NOD2 knock-out mice with C57BL/6 background	N/R	GSK669	NOD2 antagonist
Reiner et al^41^	2015	Mouse	C57BL/6	N/R	Alpha-linolenic acid	Omega-3 fatty acid
Yao et al^51^	2019	Rats	Sprague–Dawley	N/R	Resveratrol	SIRT1 agonist
Liu et al^53^	2019	Rats	Sprague-Dawley	Eight in each group	Danhong Huayu Koufuye	Inhibiting inflammation via SIRT1/NF-κB signaling pathway
Lu et al^54^	2021	Rats	Sprague-Dawley	56	Paeoniae radix rubra	Inhibiting GSK3β activity
Yadav et al^47^	2019	Mice	Unspecified; genotype controls with a heterozygous CD39 knockout	11	Knockout of ENTPD-1	Vascular ectoapyrase

CO, Carbon monoxide; *DVT*, deep vein thrombosis; *EGCG*, epigallocatechin gallate; *IP*, intraperitoneal; *IV*, intravenous; *NO*, nitric oxide; *NR*, not reported; *NSAID*, non-steroidal anti-inflammatory drugs; *PE*, pulmonary embolism; *PO*, oral.

**Table II:** Overview of experimental methodologies from the included studies

Author	Year	Model	Condition	Treatment	Class	Route	Timing	N
Zhang et al^52^	2016	Stasis ligation	DVT	DHK	TCM	PO	1 hour post-op × 7 days	48
Qi et al^36^	2017	Stasis ligation	DVT	Colchicine	NSAID	IP	Daily	60
Kessinger et al^44^	2015	Stasis ligation	DVT	Statins	Statin	PO	Unstated	282
Wang et al^35^	2017	Stasis ligation	DVT	Methotrexate	Selectin inhibitor	IP	Daily	60
Li et al^42^	2016	Stasis ligation	DVT	NaHS	eNOS/iNOS ↑	IP	Unstated	30
Zhang et al^71^	2017	Thrombus injection	PE	Aspirin	NSAID	IV	One dose, 3 days pre-op	90
Zhou et al^43^	2020	Stasis ligation	DVT	TJ2	TCM	PO	Daily × 14 days	16
Anyanwu et al^33^	2012	Stasis ligation	DVT	CO	Selectin inhibitor	INH	7 days	12
Bedard et al^49^	2008	FeCl injection	DVT	PSI-697	Selectin inhibitor	PO	Unstated	15
Chen et al^39^	2019	PE model	PE	Catestatin	Peptide	IV	Unstated	80
Chen et al^73^	2022	Stasis ligation	DVT	Rg5	Ginseng derivative	Unstated	Unstated	48
Dai et al^74^	2022	IVC stenosis	DVT/PE	Ruscogenin	Steroid	IG	Unstated	30
DeRoo et al^32^	2012	Stasis ligation	DVT	Rosuvastatin	Statin	Unstated	Unstated	Unstated
Diaz et al^34^	2009	Stasis ligation	DVT	ARC5690	Selectin inhibitor	IP	Unstated	Unstated
Feng et al^65^	2017	IVC stenosis	DVT	Simvastatin	Statin	PO	Unstated	48
Henke et al^50^	2000	Stasis ligation	DVT	IL-10	Cytokine	IV	Viral Tx preligation	4
Myers et al^57^	2002	Balloon	DVT	rPSGL-Ig	Selectin Inhibitor	IV	×14 days postligation	8
Neto-Neves et al^70^	2011	Autologous clot	PE	Atorvastatin + sildenafil	Statin + NO	Mixed	Week pre +30 minutes post	22
Pan et al^40^	2021	Collagen/Epi injection	PE	GSK669	NOD2 antagonist	IV	15 minutes pre	Unstated
Patterson et al^37^	2013	Stasis ligation	DVT	Rosuvastatin	Statin	PO	Daily pre-op	180
Reiner et al^41^	2015	IVC stenosis	DVT	ALA	Omega-3 FA	PO	4 weeks pre	Unstated
Rossiello et al^38^	2005	IV collagen/Epi	PE	Nitropravastatin	Statin/NO	IP	3 hours pre	Unstated
Souza-Costa et al^48^	2007	Perfusion	PE	Atorvastatin	Statin	PO	2 weeks pre	Unstated
Sun et al^72^	2020	IVC stenosis	PE	Unspecified steroid	Steroid	IV	Unstated	40
Yao et al^51^	2019	Stasis ligation	DVT	Resveratrol	SIRT1 agonist	PO	1 hour pre +24 hours post	Unstated
Myers et al^58^	2020	Balloon	DVT	GMI-1271	Selectin inhibitor	SC	Daily × 21 days	9
Lu et al^54^	2021	Stasis ligation	DVT	PRRDG	GSK3β inhibitor	PO	Unstated	56
Meier et al^62^	2008	Stasis ligation	DVT	PSI-421	Selectin Inhibitor	PO	Unstated	9

ALA, alpha-linolenic acid; CO, carbon monoxide; DHK, Danhong huayu koufuye; DVT, deep vein thrombosis; Epi, epinephrine (adrenaline); eNOS, endothelial nitric oxide synthase; FA, fatty acids; FeCl₃, ferric chloride; GSK3β, glycogen synthase kinase 3 beta; IG, intragastric; IL, interleukin; IP, intraperitoneal; INH, inhalation; iNOS, inducible nitric oxide synthase; IV, intravenous; IVC, inferior vena cava; NaHS, sodium hydrosulfide; NO, nitric oxide; NOD2, nucleotide-binding oligomerisation domain-containing protein 2; NSAID, nonsteroidal anti-inflammatory drug; PE, pulmonary embolism; PO, per os (oral administration); PRRDG, Paeoniae Radix Rubra dispensing granule; rPSGL-Ig, recombinant P-selectin glycoprotein ligand–immunoglobulin fusion protein; SC, subcutaneous; SIRT1, sirtuin 1; TCM, traditional Chinese medicine; TJ2, Tongji 2.

**Table III:** Overall summary of the results from the included studies

Author	Timing	Thrombus	Fibrosis	Inflammation
Zhang et al^52^	7 days	DHK ↓ thrombus by 49%, ↑ recanalization	↓ Vein wall thickness	↓ NEUT, LYM, TNF-α, MMP-9, TF; no effect on IL-6
Qi et al^36^	Day 4/8	No significant difference	↓ Vein wall scarring	↓ F4/80+, FSP1+ cells, IL-1β, CD68
Kessinger et al^44^	10 days	Atorvastatin ↓ thrombus	↓ Scarring (day 8+)	↓ Inflammation (day 4), Mac-3 ↑
Wang et al^35^	Day 4/8	No significant difference	↓ Vein wall thickness	↓ Macrophages, CD68, cathepsin B
Li et al^42^	48 hours	NaHS ↓ thrombus	N/A	↑ eNOS, iNOS; L-NAME reversed
Zhang et al^71^	6 hours	“Good thrombolysis”	N/A	↓ HR, PAP; ↑ inflammation
Zhou et al^43^	2 weeks	TJ2 ↓ thrombus weight	N/A	N/A
Anyanwu et al^33^	7 days	CO_2_ ↓ thrombus	Not reported	↓ IL-1β, IL-6, TNF-α, CCL-2
Bedard et al^49^	1-2 hours	PSI-697 ↓ thrombus (18%)	Not reported	Not reported
Chen et al^73^	10 min/12 h	Catestatin ↑ survival; Rg5 ↓ thrombus	N/A	↓ IL-6, TNF-α, IL-1β
Dai et al^74^	24-96 hours	RUS ↓ thrombus	N/A	↓ IL-6, IL-1β
DeRoo et al^32^	3 hours-2 days	Rosuvastatin ↓ thrombus	N/A	↓ IL-6, CRP, TNF-α, PAI-1
Diaz et al^34^	3 days	↓ Thrombus	N/A	↓ leukocyte, neutrophil counts
Feng et al^65^	3-14 days	Simvastatin + heparin ↓ thrombus	N/A	↓ IL-6, MCP-1, P-selectin
Henke et al^50^	4 days post-tx	No effect on clot	N/A	↓ P/E-selectin, ICAM-1
Myers et al^57^	16/90 d	rPSGL-Ig ↑ reopening	Not reported	↓ Inflammation (qualitative)
Pan et al^40^	5 hours post-PE	↓ Thrombi (figures)	N/A	↓ MDA, ↑ SOD
Patterson et al^37^	3 hour-2 days	↓ Thrombus (day 2)	N/A	↓ sPsel, PAI-1, IL-6
Riener et al^41^	N/R	No effect	Not reported	Not reported
Rossiello et al^38^	15 minutes	N/R	N/R	↓ TF expression in vitro
Souza-Costa et al^48^	Not reported	N/R	N/R	Atorvastatin ↓ MMP-9 (lung)
Sun et al^72^	3 hour-7 days	Not reported	Not reported	↓ IL-1, IL-6, TNF-α, NETs
Trejo et al^68^	20 minutes	Not reported	Not reported	↓ Edema
Yao et al^51^	0.5 hour-14 days	RES ↓ thrombus	N/R	↓ IL-1β, TNF-α
Lu et al^54^	7 days	PRRDG ↓ thrombus (59%-75%)	N/A	↓ TNF-α, IL-1β
Liu et al^53^	48 hours	DHK ↓ thrombus (38%)	N/A	↓ IL-1β, TNF-α; ↑ SIRT1
Meier et al^62^	6 days	PSI-421 ↑ patency	N/A	↓ Inflammation (MRV)
Wakefield et al^63^	2-30 days	rPSGL-Ig ↓ thrombosis	N/A	↓ IL-8, MCP-1, vein inflammation
Wang et al^56^	Day 2/8	NO microbubbles ↓ thrombus	N/A	↓ platelet + inflammatory cell adhesion
Yadav et al^47^	Not reported	CD39 KO ↑ thrombosis	N/R	IL-1β neutralization ↓ inflammation

CCL-2, C-C motif chemokine ligand 2; CD68, cluster of differentiation 68; CO, cardiac output; CRP, C-reactive protein; DHK, Danhong huayu koufuye; eNOS, endothelial nitric oxide synthase; FSP1, fibroblast-specific protein 1; HR, heart rate; ICAM-1, intercellular adhesion molecule 1; IL, interleukin; iNOS, inducible nitric oxide synthase; KO, knockout; L-NAME, Nω-nitro-L-arginine methyl ester; LYM, lymphocytes; Mac-3, macrophage antigen 3; MCP-1, monocyte chemoattractant protein-1; MDA, malondialdehyde; MMP-9, matrix metalloproteinase-9; MRV, magnetic resonance venogram; N/A, not applicable; NETs, neutrophil extracellular traps; NEUT, neutrophils; NO, nitric oxide; N/R, not reported; PAI-1, plasminogen activator inhibitor-1; PAP, pulmonary arterial pressure; PE, pulmonary embolism; RES, resveratrol; RUS, ruscogenin; SIRT1, sirtuin 1; SOD, superoxide dismutase; sPsel, soluble P-selectin; TF, tissue factor; TNF-α, tumor necrosis factor alpha.

Sex-specific analyses were rarely reported (Table I). Among the included studies, most either used exclusively male animals or did not report the sex of the animals studied. None of the included studies performed sex-stratified analyses of therapeutic outcomes. The timing of therapeutic administration varied substantially across studies (Table II). Several studies initiated treatment before thrombosis induction (eg, days to weeks before model creation), whereas others administered therapy immediately after or shortly following thrombosis induction. However, many studies did not clearly report treatment timing. Pretreatment approaches were particularly common among studies evaluating statins and anti-inflammatory compounds. Fibrosis outcomes were reported in only a minority of included studies and were assessed using heterogeneous methodologies (Table III). Reported measures included vein wall thickness, histologic scarring, and qualitative vein wall remodeling. No standardized fibrosis assessment methods were used across studies, and quantitative collagen measurements or standardized histologic scoring systems were rarely reported.

### Venous fibrosis

Six studies and two conference abstracts reported measures of venous fibrosis outcomes, all within DVT models.^35^^,^^36^^,^^44^^,^^45^^,^^52^^,^^58^^,^^59^^,^^64^ However, a meta-analysis for the primary outcome was not possible because of a lack of sufficiently similar outcome reporting methods. A key limitation is the heterogeneity in fibrosis outcome reporting, including differences in histologic techniques, quantification methods, and time points assessed. This lack of standardization impedes direct comparisons across studies. Future preclinical studies would benefit from adopting a core outcome set for fibrosis in VTE models—such as a consistent use of vein wall thickness, collagen staining, or scoring systems validated against human pathology—to improve reproducibility and translational relevance.

#### Statins

Two studies conducted in mice reported fibrosis outcomes after thrombosis and statin administration. Kessinger et al^45^ tested oral rosuvastatin against phosphate-buffered saline (PBS), administered on day −1. They demonstrated a 43% reduction in venous fibrosis, as measured by vein wall collagen thickness, with oral rosuvastatin compared with the PBS control (PBS: 56.2 ± 14.8 μm [n = 12] vs statin: 32.0 ± 9.41 μm [n = 8]; *P* = .0150). Kessinger et al^44^ tested oral atorvastatin against the PBS for vein wall collagen thickness, administered via oral gavage 24 hours after DVT induction. They showed that scarring was decreased by 43.9%, 39.0%, and 47.6% at days 8, 12, and 21, respectively, with statin compared with PBS-treated animals (*P* < .0001). As there were only two studies, and the number of animals was not reported in one of them, a meta-analysis could not be performed.

#### Selectin inhibitors

Specific selectin inhibitors and the antimetabolite drug methotrexate, which is known to reduce E-selectin expression,^75^ were included in this group. Four studies on selectin inhibitors reported fibrosis outcomes: three in baboons and one in mice. Myers et al^59^ assessed the small molecule E-selectin inhibitor GMI-1271 in baboons (primates), using a single blinded pathologist to score histological specimens for intimal thickness and fibrosis. The E-selectin inhibitor, administered on the second day following induction of thrombosis, significantly decreased intimal thickness compared with untreated baboons (*P* < .001; n = not stated) and those receiving LMWH as a control (*P* < .001; n = not stated). Myers et al^58^ also tested GMI-1271 in baboons (primates), given on postocclusion day 2, using the same pathologic scoring system for fibrosis. GMI-1271 significantly decreased intimal thickness (*P* < .001; n = not stated) and intimal fibrosis (*P* < .05; n = not stated) compared with untreated controls. In addition, P-selectin inhibition has been shown to promote thrombus resolution and reduce vein wall fibrosis in experimental models, supporting the potential dual benefit of targeting leukocyte-endothelial interactions in venous thrombosis.^64^ Lastly, Wang et al^35^ published a conference abstract comparing daily intraperitoneal injections of methotrexate (from day 1 to sacrifice) with PBS. Methotrexate reduced vein wall collagen thickness by 36.52% compared with PBS at day 8 (*P* < .05; n = 6 per group).

#### Antimitotic agents

One study, published as a conference abstract, assessed the efficacy of an NSAID, colchicine, for venous fibrosis.^36^ Both low- and high-dose colchicine were compared with PBS, and the outcome measure used was vein wall collagen thickness. Outcomes were measured on days 8 and 14. On day 8, vein wall collagen thickness was 71.53 ± 6.97 μm with PBS, 55.11 ± 5.48 μm with low-dose colchicine (Col lo) and 46.84 ± 2.03 μm with high-dose colchicine (Col hi) (PBS vs Col lo, *P* < .01; Col lo vs Col hi, *P* < .05; n = 5 per group). On day 14, vein wall collagen thickness was 55.29 ± 8.97 μm with PBS, 45.20 ± 2.73 μm with Col lo, and 42.68 ± 3.57 μm with Col hi (PBS vs Col lo and Col hi, *P* < .05; n = 4-7 per group). In addition, colchicine reduced RNA expression of profibrotic/fibrotic markers (collagen I, collagen III, procollagen I, procollagen III, and FSP1) (n = 8 per group; *P* < .05).

#### Other anti-inflammatory agents

One other agent assessed for its ability to reduce venous fibrosis was Danhong Huayu Koufuye (DHK), a traditional Chinese medicine.^52^ According to the study, it contains 29% *Salvia miltiorrhiza* radix, 11.5% *Angelicae sinensis* radix, 15% Chuanxiong rhizoma, 11.5% Persicae semen, 11.5% Carthami flos, 11.5% Bupleuri radix, as well as 10% Aurantii fructus, and is thought to be anti-inflammatory. The study was conducted in rats, which were administered DHK or warfarin orally. DHK significantly reduced the vein wall thickness by 47.86% as compared with warfarin (134.56 ± 31.66 mm vs 258.06 ± 22.56 mm; *P* < .01; n per group not reported).

### Thrombosis

Thirty-five identified articles reported measures of venous thrombosis outcomes. Measures included, but were not limited to, thrombus burden, resolution, and weight reduction, as well as venous recanalization. The outcomes have been summarized for the main drug classes below.

#### Statins

Kessinger et al^44^ examined whether statins reduce thrombus burden and vein wall scarring in established venous thrombosis using C57/BL6 mice (n = 282). The atorvastatin-treated animals exhibited a reduced inferior vena cava (IVC) thrombus burden at days 4, 7, and 10 relative to controls. In addition, accelerated early venous thrombus resolution to a greater extent was observed in the atorvastatin group.^44^ Kessinger et al^45^ explored the same hypothesis but with rosuvastatin in C57BL/6J mice. They found a 39% decrease of in venous thromboembolism (VT) as measured by fluorodeoxyglucose positron emission tomography (FDG-PET) mean standardized uptake values (SUVmean) in statin-treated animals (n = 8) compared with those treated with PBS (n = 12) (*P* = .017). Statin therapy also decreased the maximum standardized uptake value (SUVmax) and target-to-background values in day 2 venous thrombi. The SUVmax in the PBS group was 1.82 ± 0.41 compared with that in the statin therapy group, which had an SUVmax of 1.06 ± 0.27 (*P* = .0246). The target-to-background value for PBS was 2.65 ± 0.77, and for statin therapy, it was 1.66 ± 0.46 (*P* = .019). The surgical sham animals (n = 5) showed low levels of FDG uptake or no uptake in the IVC.^45^

DeRoo et al^32^ looked at the effects of rosuvastatin on venous thrombosis in a mouse model of DVT. Although the study recorded thrombus weight as nonsignificant at the 3- and 6-hour points, this was found to be significantly decreased at day 2 after DVT in the rosuvastatin group (0.025 ± 0.001) compared with the control (0.033 ± 0.001) (*P* < .05).^76^

Feng et al^65^ analyzed simvastatin for thrombus burden and venous inflammation. The study used New Zealand white rabbits with n = 4 per group. A significant drop in thrombus weight/length ratio was recorded with combined treatment of simvastatin and heparin from day 3 until day 12 (*P* < .05 compared with controls). The reduction was also greater with simvastatin compared with heparin at day 7, but not at other time points (*P* < .05).^65^ Feng performed a similar study a year prior using simvastatin for venous thrombus burden and resolution compared with control.^72^ New Zealand white rabbits were randomly divided into four groups: control, simvastatin, LMWH, and LMWH + simvastatin. The study found that the values in the simvastatin group, LMWH group, and LMWH + simvastatin group were significantly lower than those in the control group on days 3, 7, and 14 (*P* < .05).^66^

Patterson et al^37^ examined the impact of rosuvastatin on DVT in hyperlipidemic mice with ApoE gene-deleted mice. There was no significant difference found between the intervention and the control group (saline) at the 3- or 6-hour point in thrombus weight. After 2 days, there was a statistically significant decrease in the rosuvastatin group compared with the control group (0.0304 ± 0.0010 vs 0.0346 ± 0.0011 g; *P* < .05).^37^

#### Antimitotic agents

One conference abstract published by Qi et al^36^ assessed the effect of colchicine on venous scarring, which reported there was no impairment to VT resolution when measuring it as a thrombus mass, which was similar between the groups at days 4 and 8.

#### Nonsteroidal anti-inflammatory drugs

Zhang et al^71^ investigated the role of NSAIDs in thrombosis, using aspirin in Sprague-Dawley rats with PE through injection of autologous thrombus into the jugular veins. The authors administered 300 mg/kg of aspirin intravenously 3 days before the intervention and performed histologic assessment at 6 hours from modeling. This revealed evidence of significant TXA2 reduction, which translated to positive thrombolytic effects and endothelial hyperplasia.^71^

#### Selectin inhibitors

Ten studies reported measures of thrombosis and selectin inhibitors. Myers et al, 2022^59^ and Myers et al, 2020,^58^ both assessed the role of E-selectin in the treatment of experimental venous thrombosis. Both studies reported significantly improved vein recanalization, assessed using magnetic resonance venography (MRV), as well as significantly reduced clot strength, measured thromboelastographically, at several time points in animals treated with GMI-1271 compared with control and comparator groups (*P* < .05).^58^^,^^59^ In addition, an ultrasound analysis was conducted following this by Myers et al,^59^ which looked at three segments of the lumen. Of the four groups, the open vein lumen returned to baseline levels only in the GMI group (+6%). The control group had an 80% (−80%) open vein lumen decrease, the GMI + LMWH group had a 26% (−26%) decrease, and the LMWH group showed a 27% (−27%) decrease.^59^ In Myers et al,^58^ animals in the GMI-1271 group had a significant increase in the right iliac open lumen area compared with the control groups (one-way analysis of variance, *P* < .001; control vs GMI-1271 area, *P* < .05). However, the iliac vein valve reflux at day 21 showed no significant differences between GMI-1271 and control groups using Fisher exact test (*P* = .017).^58^

Myers et al^57^ used another P-selectin antagonist (rPSGL-Ig) administered intravenously in male *Papio anubis* (n = 3), whereas another group received LMWH (n = 2). A control group received normal saline (n = 3). Statistically significant differences in spontaneous vein reopening were noted for animals treated with LMWH and rPSGL-Ig compared with the control group. This was evident at day 16 (*P* < .05) in the proximal iliac vein and at day 9 (*P* < .01) in the midiliac vein.^57^

Anyanwu et al^33^ published a conference abstract that the use of a P-selectin inhibitor decreased thrombus mass in a murine model of venous thrombosis. The P-selectin inhibitor, carbon monoxide (CO), resulted in a significant decrease (n = 5; *P* < .05) in the thrombus weight in C57BL/6 mice compared with the control group. Bedard et al^49^ used the P-selectin inhibitor (PSI-697) in rats and measured the thrombus burden. Results showed that thrombus weights overall were reduced by 18% (*P* < .05). There was also no increase in the duration of bleeding time.^33^

Diaz et al^34^ used ARC5690, a P-selectin inhibitor, to assess its effects on venous inflammation after venous thrombosis. Three dosage groups relating to ARC5690 were used in the study: 2, 5, and 10 mg/kg. Thrombus weight was significantly decreased in the 5 mg/kg group compared with the ligation only (LO) group and the anti-P-selectin control aptamer (PSCA) 1 mg/kg group (*P* < .05). In another group with anti-P-selectin antibody (APSB 0.2 mg/kg IP) in the study, there was a significant decrease in thrombus weight compared with the LO group and the PSCA group (1 mg/kg and 10 mg/kg). A positive correlation was observed between thrombus weight and plasma soluble P-selectin, with a Spearman correlation value of 0.55.^34^

Meier et al^62^ used PSI-421 (P-selectin inhibitor) in Papio anubis. Three groups were formed: those that received PSI-421, those receiving enoxaparin, and a control group. Prophylactic enoxaparin and prophylactic PSI-421 promoted vein lumen patency. At day 6, the PSI-421 group had greater vein lumen patency than those that received enoxaparin (59% ± 20% vs 38% ± 15%). To note, the control animals on day 6 developed large collateral veins and had 0% reopening through day 6.^62^

Myers et al^60^ used rPSGL-Ig on Papio anubis, exploring the effect of P-selectin inhibition on inflammation and thrombosis. Four groups were established: control, 500 μg/kg, 1 mg/kg, and previously reported 4 mg/kg. The primary outcome was the presence or absence of thrombus in a venous segment. This was evaluated at two time points from baseline and in different sites of multiple vein segments. Upon assessing the proportion of segments with thrombus at the level of the mid-IVC, results showed a significant dose-response relationship (*P* < .01).^60^

Myers et al^61^ looked at the effect of P-selectin inhibition on the resolution of venous thrombosis using PSI-697. At the 6-hour time point (balloon is deflated), all primates in the control group and PSI-697 animals demonstrated large thrombi by ultrasound. The level of vein opening was significantly increased in the PSI-697 animals compared with the control group using MRV at day 2 (78% ± 10% vs 0% ± 0%; *P* < .01) and day 6 (78% ± 8% vs 0% ± 0%; *P* < .01).^61^

Wakefield et al^63^ demonstrated the impact of P-selectin inhibition on thrombosis and inflammation parameters in their study. Their results showed significantly decreased IVC thrombosis in rPSGL-Ig-treated animals (1/16; 6%) compared with the controls (5/8; 63%; *P* < .01). In the period from day 2 to day 6, there was a significant increase in spontaneous thrombosis resolution, including at balloon sites, in the rPSGL-Ig animals compared with control animals (23% vs 2%; *P* < .001), without PE.^63^

A unique pegylated P-selectin inhibitor, P-G6, was trialed by Wong et al,^77^ in C57BL/6 mouse models. This demonstrated inhibition of P-selectin and PSGL-I binding, with decreased platelet-leukocyte aggregation in vitro and in vivo. In addition, a clear reduction in thrombus burden was observed. Importantly, this occurred without negative effects on hemostasis, particularly bleeding time.^77^

Wang et al^35^ published a conference abstract on the impact of methotrexate on vein wall scarring in DVT on C57BL/6 mice. Thrombus burden, weight, length, and mass were similar for both the treatment and control groups on day 4 (*P* > .05) and day 8 (*P* > .05). The control group received PBS.^35^

#### Other anti-inflammatory agents

Dai et al^74^ published an article looking at the effects of ruscogenin (steroidal sapogenin) which was administered to C57BL/6 mice, assessing its effect on thrombus burden. The study found 1 mg/kg of ruscogenin had a significant inhibitory effect on the IVC thrombus.^78^ Reiner et al^41^ trialed two oral doses (0.03 g and 7.3 g) of alpha-lenoleic acid, an omega-3 fatty acid, also in C57BL/6 mice. However, unlike ruscogenin, alpha-lenoleic acid did not have any significant effects on thrombus incidence or size.^40^ Yadav et al^47^ investigated the impact of CD39 knockout in inflammasome-driven thrombosis, which revealed that loss of a single CD39 allele increases venous thrombosis and propagation. Li et al^42^ and Wang et al^55^ each assessed nitric oxide (NO)-based agents sodium hydrosulfide and lipidic microbubbles, respectively. Both studies reported significant reduction in thrombus size/volume compared with controls (*P* < .05).^41^^,^^54^ Traditional Chinese medicines were reported in four studies and demonstrated significantly positive effects on thrombus burden.^43^, ^52^, ^54^, ^69^

### Inflammation

All the included studies reported the effect of an anti-inflammatory agent on a measure of inflammation. Measures included, but were not limited to, local RNA expression levels of inflammatory markers, local and systemic protein expression levels of inflammatory markers, PET scanning, MRV appearance, edema formation, and inflammatory cell infiltrates (neutrophils and macrophages). The outcomes have been summarized for the four main drug classes in the following sections.

#### Statins

There were 10 studies reporting the impact of statins on inflammation: four in PE models and six for DVT. Kessinger et al^45^ showed that statins reduced IL-6 by 77% (366.2 ± 163.4 units with PBS vs 83.3 ± 78.1 units for statins; *P* = .0152) and neutrophil influx by 46% (119.3 ± 54.7 vs 64.4 ± 26.8 neutrophils per high power field for PBS vs statin treatment; *P* = .0055; n = 5-6 per group), at day 2 compared with PBS-treated animals. Inflammation measured by PET computed tomography standardized uptake value predicted venous fibrosis at day 14.^45^ DeRoo et al^32^ published a conference abstract which showed that statins reduced plasminogen activator inhibitor-1 (PAI-1), IL-6, C-reactive protein, and tumor necrosis factor alpha (TNFα) at 6 hours after DVT, as well as IL-6 and C-C motif chemokine ligand 2 (CCL2) as early as 3 hours after DVT. In a study of simvastatin vs control in rabbits, statins reduced neutrophils, monocytes, and lymphocytes in thrombus, as well as levels of IL-6, P-selectin, and monocyte chemoattractant protein-1 (MCP-1), compared with saline control (*P* < .0001 for all; n = 4 per group).^32^ Feng et al^66^ support these findings in their rabbit model of DVT by demonstrating reduced local and systemic P-selectin and PAI-1 with simvastatin at days 3, 7, and 14 compared with control animals (*P* < .05; n = not stated). Patterson et al^37^ again showed that rosuvastatin significantly decreased PAI-1 expression and plasma levels, expression of molecules related to the IL-6 pathway, and vein endothelium neutrophil migration. Kessinger et al^44^ also showed statins additionally reduced thrombus PAI-1, tissue factor, neutrophils, myeloperoxidase, neutrophil extracellular traps, and macrophages, and these effects were most notable at earlier time points after DVT formation. In PE models, statins reduced TNFα,^67^ IL-1β,^67^ and MMP9.^48^ Pretreatment with atorvastatin prior to PE led to reduced MMP2 but not MMP9.^70^

#### Antimitotic agents

The aforementioned Qi et al^36^ administered 0.2 or 0.02 mg/kg of colchicine in C57BL/5 mice DVT models and assessed inflammation at days 4, 8, and 14. This revealed a reduction in the number of vein wall F4/80+ macrophages (55.87 ± 8.07; *P* < .05) and FSP1+ cells (41.60 ± 8.37; *P* < .01). Also, colchicine reduced RNA expression of both profibrotic and proinflammatory markers (*P* < .05), without impairing thrombus resolution.^36^

#### Nonsteroidal anti-inflammatory drugs

Two studies assessed the efficacy of NSAIDs for venous inflammation, both performed on Sprague-Dawley rats using autologous thrombus injection into the jugular vein. When given 40 minutes before modeling, Zhang et al^71^ recorded a significant decrease in heart rate, pulmonary artery pressure (PAP), and pulmonary arterial diastolic pressure (*P* < .05) compared with both the control and model groups. In contrast, Wang et al^56^ reported a significant drop in the levels of extracellular signal-regulated kinase, phosphoinositide 3-kinases, and protein kinase B in the control, sham, and the three aspirin groups at 6-, 24-, and 72-hour time points (*P* < .001 or *P* < .01).

#### Selectin inhibitors

Seven studies and three conference abstracts investigated the relationship between selectin inhibitors and venous inflammation. The latter all used C57BL/6 mice to test different selectin inhibitors in a stasis-ligation DVT model. Wang et al^35^ injected 0.7 mg/kg of methotrexate daily and subsequently observed inhibition of vein wall mRNA expression of inflammatory markers CD68 and cathepsin B (*P* < .05 vs PBS). In addition, the authors reported inhibition of macrophage recruitment in the vein wall at day 8 (*P* < .05 vs PBS).^35^ Anyanwu et al,^33^ on the other hand, administered 50 ppm of CO for a period of 7 days, which resulted in decreased expression of proinflammatory cytokines (IL-1beta, IL-6 and TNF-alpha, CCL-2) in the vein wall and thrombus (n = 4; *P* < .001), relative to normotoxic mice. Diaz et al^34^ studied the effects of aptamer ARC5690 on venous inflammation following acute DVT. Histologic analysis revealed a significantly reduced leukocyte count in the treated groups (5 mg/kg and 10 mg/kg) relative to the control and 2 mg/kg treated groups (*P* < .05). The same result was recorded for neutrophils, monocytes, and lymphocytes.^33^

The remaining seven studies were in baboons. Myers et al^60^ and Myers et al^57^ looked at the selectin inhibitor rPSGL-Ig. The first study administered 500 μg/kg (n = 3) and 1 mg/kg (n = 5) rPSGL-Ig; yet, no dose-response relationship was found when assessing cytokine levels. Evaluation of vein wall cytokine levels revealed lower levels of IL-8 and MCP-1 with rPSGL-Ig treatment in both the left and right iliac veins, as well as the IVC (*P* < .05 [IL-8] by ANOVA; *P* < .01 [MCP-1] by ANOVA) with no real differences in PF-4 (all noncorrected to total protein). Cytokine values corrected to total protein revealed similar results. Finally, morphometric analysis demonstrated no significant differences in inflammatory cell extravasation between groups.^60^ As for Myers et al,^57^ the treatment resulted in reduced vascular inflammation; however, the effect did not reach significance because of the small sample size (n = 8).

Wakefield et al^63^ also studied rPSGL-Ig in 12 juvenile baboons using endovenous balloon-induced stasis; 4 mg/kg was injected intravenously 1 hour before the cut downs were performed. MRV showed significantly reduced inflammation, as well as IL-8 and MCP-1 levels, at days 2 and 6. However, no significant difference was found between groups in the number of inflammatory cells.^63^

GMI-1271 was used in both Myers et al, 2020,^58^ and Myers et al, 2022.^59^ The prior study gave nine subjects 25 mg/kg of subcutaneous GMI-1271 for 21 days. MRV revealed a significant reduction in vein wall inflammation compared with control on day 14 (*P* < .0001) and day 21 (*P* < .0001). However, no sufficient effect on vein wall inflammatory cell extravasation (migration) was observed.^58^ In Myers et al,^59^ the GMI-1271-treated group showed significantly decreased vein wall inflammation by MRV with gadolinium (Gd) vein wall enhancement compared with controls on day 21 (*P* < .0001). A different selectin inhibitor, PSI-697, showed similar results in Myers et al.^61^ At 6 days after administration of 30 mg/kg, the authors recorded a significant decrease in vein wall inflammation on MRV compared with controls (11 ± 3 mm^2^ Gd enhancement vs 28 ± 4 mm^2^ Gd enhancement; *P* < .01).^61^

#### Steroids

The aforementioned Dai et al^74^ showed that following the intragastric administration of 0.1 mg/kg ruscogenin in C57BL/6 mice, the expression of IL-6 and IL-1β was substantially inhibited.^78^ Sun et al^72^ administered 0.1 to 0.25 μM of glucocorticoid in 40 BALB/c mice PE models and observed considerable inhibition of IL-1, IL-6, and TNF-α. The level of inhibition was shown to be directly correlated to the steroid concentration. In addition, glucocorticoid-treated neutrophils produced fewer neutrophil extracellular traps and had reduced inhibition of NF-kB.^72^ Using 30 rabbits in which autologous thrombus was directly injected into the pulmonary artery, Trejo et al^68^ investigated the impact of hydrocortisone (200 mg) and papaverine on lung tissue after thromboembolism. Although hydrocortisone had a more positive effect, the authors suggested that it could not prolong the viability of the preparation as papaverine did for 20 minutes after embolism.^68^

#### Other anti-inflammatory agents

The aforementioned Dai et al,^74^ showed that 0.1 mg/kg of the steroidal sapinogen ruscogenin considerably inhibited the mRNA expression of IL-6 and IL-1β.^78^ The aforementioned vascular ectoapyrase study of ENTPD-1 knockout by Yadav et al^47^ observed exaggerated inflammasome activation in venous thrombosis in CD39-deficient mice. NO-based agents, sodium hydrosulfide and lipidic microbubbles, in Li et al and Wang et al, proved their positive effects on inflammation, showing higher endothelial nitric oxide synthase and inducible nitric oxide synthase protein expression with the former and attenuated aggregation of platelets and inflammatory cells with the latter.^42^^,^^55^ Three traditional Chinese medicines were studied by Li et al^69^ (tea polyphenol epigallocatechin-3-gallate), Lu et al^54^ (*paeoniae radix rubra*), and Zhang et al^52^ (DHK). All three agents resulted in improved inflammation through the reduction of a variety of inflammatory cells.^51^^,^^68^

### Risk of bias

Risk of bias assessment was conducted using the SYRCLE quality assessment tool. All the studies included in the review were found to be at unclear or high risk of bias, and as such, the certainty of evidence presented by this review is very low.

## Discussion

The current systematic review studied the role of anti-inflammatory treatments, namely statins, NSAIDs, selectin inhibitors, and steroids among others, in preclinical models of venous thromboembolism, focusing on venous fibrosis, thrombosis, and inflammation. Overall, broad anti-inflammatory classes have emerged from the analysis for evaluation: statins, selectin inhibitors, steroids, NSAIDs, and antimitotic agents.

Statin therapy has been shown to reduce the occurrence and progression of atherosclerosis, leading to improved outcomes for myocardial infarction, stroke, composite cardiovascular diseases, and all-cause mortality.^79^ Moreover, statins appear to have a benefit in VTE, reducing the risk of DVT recurrence by 33% in meta-analysis.^80^ In animal models of VTE, statins have reduced vein wall scarring by 40% to 43% without impairing thrombus resolution.^44^^,^^45^ Critically, one of the studies showed that reductions in inflammation at day 2 predicted improvements in venous fibrosis.^45^ Kessinger et al^44^ can be considered one of the largest animal studies in the literature investigating the effect of statins on thrombus burden and vein wall scarring in established venous thrombosis. This showed the role of atorvastatin in reducing all of fibrosis, thrombosis, and inflammation.^44^ Similar positive effects on venous thrombus and inflammation were seen with rosuvastatin in Patterson et al.^37^ However, no significant differences were recorded in cholesterol and triglyceride levels between mice treated with rosuvastatin compared with saline.^37^

Perhaps, the best-studied class of anti-inflammatory agents in VTE is the selectin inhibitors. Selectins are cellular adhesion molecules that modulate the recruitment of inflammatory cells to thrombus and vein wall. Two studies of endothelial (E)-selectin inhibitors reduced vein wall fibrosis in a baboon model of DVT, resulting in improved thrombus resolution and reduced measures of inflammation, superior to that of heparin without prolonging bleeding time.^58^^,^^59^ Platelet (P)-selectin inhibitors have also shown significant promise in reducing venous inflammation and thrombus burden, although studies of fibrosis are less common.^60^^,^^61^^,^^63^^,^^64^ Moreover, selectin inhibitors have been shown in a large animal study to reduce vein wall thickness by nearly 37%.^35^^,^^57^, ^58^, ^59^, ^60^ Their role in the inhibition of macrophage recruitment of vein wall mRNA expression of inflammatory markers has also been recorded in the literature. However, a study on methotrexate reported no significant effect on thrombus burden, weight, length, and mass.^35^ Importantly, the selectin inhibitors in the included studies are not licensed for clinical use in humans, although one such agent that does show reduced cellular adhesion molecule expression, including E-selectin, is methotrexate.^75^ That being said, Devata et al^81^ conducted the first-in-human study of GMI-1271. This was administered to healthy volunteers to assess its safety, tolerability, and pharmacokinetic properties and to two patients with DVT to measure biomarkers of hemostasis. Focusing on the latter group, both patients exhibited accelerated recovery after 48 hours of drug administration, with repeat ultrasound study showing signs of DVT resolution.^81^

Steroids are immunomodulating agents frequently used to reduce acute or chronic inflammation and prevent subsequent tissue damage in various contexts, including coronavirus disease 2019 (COVID-19), rheumatoid arthritis, allergies, inflammatory bowel disease, and asthma. The anti-inflammatory actions of steroids are mediated through the effect of glucocorticoid activity on transcription factors of proinflammatory cytokines, including AP-1, NF-κB, NFAT, STAT-6, and STAT-4.^76^ Documented effects are seen on IL-1, IL-2, IL-4, IL-5, IL-6, IL-12, IFN-γ, and TNFα.^76^ All immune cells are affected, with core changes such as macrophages and monocytes engaging in reduced apoptosis and release of inflammatory mediators, reduced activation of T-cells and reduced production of cytokines such as interferon by dendritic cells, reduced neutrophil activation, adhesion, and rolling.^82^ Although there are no in-human studies of steroids for VTE, they have been demonstrated to have a marked effect in other contexts; inhaled glucocorticoids in asthma are used to control chronic airway inflammation and progression, exhibiting their effect via IL-4 and IL-13 among others, two profibrotic cytokines previously discussed. COVID-19 was linked to venous thromboembolism and, fascinatingly, many parallels exist between the two disease states through systemic AhR activation syndrome consisting of inflammation, thromboembolism, and fibrosis. Low-dose steroids have famously been used after evidence from the RECOVERY trial, which demonstrated a 35% reduction in patients with intensive treatment unit (ITU) requiring invasive ventilation, the mechanism for which is thought to be steroid-dependent reduction in fibroblast proliferation, inflammatory cell exudation, reduced tissue damage, and decreased cytokine production.^83^ Steroids protect the venous endothelium from proinflammatory activation, indicating that they may be able to modulate inflammation in the context of thrombosis.^84^ Steroids have been used in PE models, resulting in significant improvements in reactive oxygen species formation and inflammatory markers such as IL-1, IL-6, and TNFα and decreased edema in parenchymal tissue.^68^^,^^72^

NSAIDs are cyclooxygenase inhibitors that block the synthesis of prostaglandins and thromboxane A2 produced during inflammation. Clinically, NSAIDs such as aspirin are used in cardiovascular diseases, including ischemic heart disease and stroke, for their antiplatelet effect to reduce the risk of thrombosis. Platelets may have a significant role in modulating vein wall fibrosis, with the induction of thrombocytopenia following thrombosis shown to reduce vascular smooth muscle cell accumulation and fibrosis of the vein wall in mice.^78^ The prostaglandin E2 (PGE2) pathway enhances IL-1β in subsets of T-lymphocytes and activates NF-κB synergistically with TNFα to induce fibroinflammatory cytokine production, including MCP-1.^85^ Inhibiting PGE2 signaling has previously been shown to reduce perivascular inflammation and aneurysm progression in preclinical models.^86^ The inflammasome is important in initiating thrombosis and in perpetuating chronic inflammation, with signaling through prostaglandin D2 increasing inflammasome activation and subsequent IL-1β secretion.^87^^,^^85^^,^^88^ Studies of NSAIDs in animal models of VTE, both in the context of PE, yielded reduced inflammatory cell infiltration, wall thickening, levels of inflammatory markers, and improved hemodynamics.^56^^,^^71^ The role of aspirin, in particular, was looked at in a large study of 108 Sprague-Dawley rats, which assessed alveolar wall necrosis and hemorrhage in addition to levels of extracellular signal-regulated kinase, phosphoinositide 3-kinases, and protein kinase B; all of which were significantly reduced after treatment.^55^ One conference abstract examined colchicine in a murine DVT model, revealing decreased measures of fibrosis at days 8 and 14; however, the full study was never published.^36^ Although it is commonly considered an NSAID, and although it does exhibit broad anti-inflammatory action, colchicine is now formally recognized as an independent antimitotic agent. Its mechanism of action is multifaceted and relies on preventing microtubule assembly, thereby disrupting inflammasome activation, microtubule-based inflammatory cell chemotaxis, generation of leukotrienes and cytokines, and phagocytosis.

To our knowledge, this is the first systematic review to specifically examine anti-inflammatory therapies in animal models of VTE, with a focus on post-thrombotic fibrosis. By highlighting the mechanistic link between inflammation and vein wall scarring, this review identifies a critical and previously underexplored therapeutic avenue. The strengths of our study are the comprehensive inclusion of all the literature to date studying the role of anti-inflammatory agents in animal models of VTE. Moreover, the mechanisms of these agents have been discussed in detail, and the text provides an excellent summary of the literature for scientists working in the field of VTE. Potential weaknesses include the generally low quality of studies, including conference abstracts that were never fully published or made available for clinical review. Indeed, this lack of publication of the full manuscripts may lead to a degree of publication bias, which cannot be assessed in this manuscript because of the lack of meta-analysis. Some studies showed variation in the significance of outcomes during their follow-up, which can be attributed to small sample sizes.

An important limitation of the current literature is the inconsistent evaluation of post-thrombotic fibrosis. Although fibrosis was designated as the primary efficacy outcome of this review, only a small subset of studies reported fibrosis measures, and the methods used varied substantially. Most studies relied on surrogate measures such as vein wall thickness or qualitative histologic scarring rather than standardized fibrosis quantification. The development of standardized fibrosis outcome measures for experimental venous thrombosis models would substantially improve comparability between studies and strengthen translational interpretation. Another limitation of the current preclinical literature is that many studies initiated therapy before thrombosis induction. Although this approach may provide mechanistic insight into thrombogenesis, it does not fully reflect the clinical scenario in which therapies are typically administered after thrombus formation has occurred. Future experimental studies should prioritize post-thrombosis therapeutic initiation to better model the clinical treatment of established VTE.

Although meta-analysis was planned, it was precluded by huge heterogeneity in species, studies, outcome measures, agents, doses, and poor reporting of numbers in experimental groups, which prevented proper combination. This highlights a significant weakness of animal studies vs clinical studies, where the numbers of patients are always reported. An additional limitation of the current literature is the near absence of sex-specific analyses. Many studies used only male animals or did not report the sex of the animals studied, and none evaluated differential therapeutic effects by sex. Given emerging evidence that inflammatory and thrombotic pathways may differ between sexes, future preclinical models of venous thromboembolism should incorporate sex-balanced designs and evaluate sex-specific therapeutic responses. In spite of the aforementioned limitations, we have used a narrative review to accurately characterize the current state of the literature as a platform for further study in the field.

## Conclusions

Inflammation is a catch-all term with a variety of evolutionarily conserved mechanisms leading to tissue damage and subsequent scarring. Anti-inflammatory agents, including statins, NSAIDs, and selectin inhibitors, may hold significant promise for reducing venous scarring but are yet to be compared for efficacy side-by-side. Moreover, current methodologies for preclinical models of VTE often involve anti-inflammatory agents started before the induction of thrombosis, which fails to recapitulate the clinical VTE scenario whereby patients may present post-thrombosis. In addition, and inseparably, reductions in measures of venous fibrosis are often closely linked to a reduction in the size of thrombosis. Future preclinical studies should establish the efficacy and safety of anti-inflammatory agents side-by-side after thrombus induction to tease out the potential benefits of these therapies before in-human studies.

## Author Contributions

Conception and design: BT, AD, AS

Analysis and interpretation: MJ, BT

Data collection: MJ, TBF, AG, BT

Writing the article: MJ, TBF, AG, BT

Critical revision of the article: MJ, BT, AD, AS

Final approval of the article: MJ, TBF, AG, BT, AD, AS

Statistical analysis: BT

Obtained funding: Not applicable

Overall responsibility: BT

## Funding

The work is supported by an award from the Graham-Dixon Charity and the 10.13039/501100000274British Heart Foundation for a PhD project studying the role of inflammation in the pathogenesis of post-thrombotic fibrosis.

## Disclosures

T.B.F. is employed part-time by Moderna and works part-time at a clinical trials unit. These roles were not involved in the conception, conduct, analysis, interpretation, or reporting of this systematic review.
