# Supplementary material for: Systematic review of the role of anti-inflammatory treatments in animal models of venous thromboembolism
Source: JVS Vasc Sci. 2026 Sep 21;7:100450. doi: 10.1016/j.jvssci.2026.100450 (PMC13738134; doi:10.1016/j.jvssci.2026.100450)
Supplement: Supplementary file 1 [file mmc1.docx]

**Supplementary Table 1: Overview of the Systematic Review Methodology**

| **Item** | **Description** |
| --- | --- |
| Review Registration | PROSPERO: CRD42023456793 |
| Guidelines Followed | SYRCLE and PRISMA |
| Databases Searched | MEDLINE, EMBASE, Cochrane Library (up to 3rd July 2023) |
| Search Strategy | MESH terms and free word searches; full strategy in supplement S1; no language restrictions; |
|  | Search terms: Free word and medical subject heading (MESH) terms were used |
| Grey Literature | Not screened |
| Reference Screening | Included article references screened for further studies |
| Study Selection | Two reviewers (TBF, ASG) independently screened; disputes resolved by third reviewer (BT); Endnote X20 used |
| Inclusion Criteria | Articles testing anti-inflammatory drugs; experimental studies and conference abstracts; reported at least one outcome of interest (inflammation markers, fibrosis or thrombosis parameters) |
| Exclusion Criteria | Therapies designed to exacerbate inflammation; Studies of exclusively heparin, low molecular weight heparins, direct oral anti-coagulants (hereafter known as anti-thrombotic therapies) will be excluded as these are outside the scope of the review. However, if administered in combination with an anti-inflammatory therapy the study will be eligible for inclusion; case reports, letters, opinion pieces; Not reporting a pre-specified outcome |
| Data Extraction | Standardized template in Microsoft Excel; two reviewers (TBF, ASG) independently extracted; discrepancies mediated by third reviewer (BT) |
| Data Analysis | Narrative systematic review (no meta-analysis) due to heterogeneity |
| Primary Efficacy Outcome | Impact of anti-inflammatory drugs on post-thrombotic fibrosis |
| Primary Safety Outcome | Impact of anti-inflammatory drugs on thrombus burden |
| Secondary Outcome | Impact of anti-inflammatory drugs on measures of inflammation |
| Quality Assessment | SYRCLE tool |
